# The synergism of high-intensity intermittent exercise and every-other-day intermittent fasting regimen on energy metabolism adaptations includes hexokinase activity and mitochondrial efficiency

**DOI:** 10.1371/journal.pone.0202784

**Published:** 2018-12-21

**Authors:** Antonio Real-Hohn, Clarice Navegantes, Katia Ramos, Dionisio Ramos-Filho, Fábio Cahuê, Antonio Galina, Verônica P. Salerno

**Affiliations:** 1 Max F. Perutz Laboratories, Medical University of Vienna, Vienna, Austria; 2 Laboratory of Exercise Biochemistry and Molecular Motors, Bioscience Department, School of Physical Education and Sports, Federal University of Rio de Janeiro, Rio de Janeiro, Brazil; 3 Laboratory of Bioenergetics and Mitochondrial Physiology, Institute of Medical Biochemistry Leopoldo de Meis, Federal University of Rio de Janeiro, Rio de Janeiro, Brazil; Universidad Pablo de Olavide, SPAIN

## Abstract

Visceral lipid accumulation, organ hypertrophy and a reduction in skeletal muscle strength are all signs associated with the severity of obesity-related disease. Intermittent fasting (IF) and high-intensity intermittent exercise (HIIE) are natural strategies that, individually, can prevent and help treat obesity along with metabolic syndrome and its associated diseases. However, the combinatorial effect of IF and HIIE on energetic metabolism is currently not well understood. We hypothesized that their combination could have a potential for more than strictly additive benefits. Here, we show that two months of every-other-day intermittent fasting regimen combined with a high-intensity intermittent exercise protocol (IF/HIIE) produced a synergistic effect, enhancing physical endurance (vs. control, HIIE and IF) and optimizing metabolic pathways of energy production in male Wistar rats. The IF/HIIE group presented enhanced glucose tolerance (vs. control, HIIE and IF), lower levels of plasma insulin (vs. control and HIIE), and a global activation of low Km hexokinases in liver (vs. control, HIIE and IF), heart (vs. control and HIIE) and skeletal muscle (vs. control, HIIE and IF). The IF/HIIE synergism, rather than a simply additive effect, is evidenced by increase in muscle mass and cross-section area, activation of the FoF1 ATP synthase, and the gain of characteristics suggestive of augmented mitochondrial mass and efficiency observed in this group. Finally, important reductions in plasma oxidative stress markers were present preferentially in IF/HIIE group. These findings provide new insights for the implementation of non-pharmaceutical strategies to prevent/treat metabolic syndrome and associated diseases.

## Introduction

Obesity and metabolic syndrome are both important risk factors for life threatening diseases that can target cardiovascular and hepatic systems [1, 2]. The prevalence of obesity and metabolic syndrome is a reality for developed countries, which began more than two decades ago [3]. Today, the incidence of obesity and metabolic syndrome is rapidly increasing in developing countries as well [4], leading to increased morbidity and mortality due to type 2 diabetes mellitus, non-alcoholic fatty liver disease, and cardiovascular disease [5, 6]. Recently, global climate change was implicated in the onset of obesity and type 2 diabetes due to the negative impact of higher temperatures on energy metabolism [7, 8]. This means that the overall prevalence of obesity and metabolic syndrome tends to aggravate in the next few years in pair with reduced physical activity [9].

Intermittent fasting (IF) regimens and high-intensity intermittent exercise (HIIE) are two natural strategies to prevent and mitigate obesity related diseases [10, 11]. An every-other-day IF regimen was recently demonstrated by Li, Xie [12] to dramatically reduce obesity, insulin resistance, and hepatic steatosis in rodents. The effects observed by IF were mimicked, in part, by transplantation of gut microbiota from an animal under IF to another animal with food *ad libitum*. On the other hand, the adaptations promoted by HIIE in rodents has been demonstrated to have a direct effect on the body parts recruited during the activity (e.g. skeletal muscles [13]) and an additional global effect in some organs like liver [14] and heart [15]. Both IF and HIIE approaches are appropriate treatments for obesity-related problems in humans [16, 17]. To note, IF and HIIE strategies, respectively, resemble the evolution patterns of the human diet [18], namely an erratic food availability and the high-intensity intermittent exercise analogous to hunting/gathering activities [19].

Since IF can produce different adaptations compared to HIIE, and the combination of both was recently demonstrated to increase skeletal muscle mRNA levels of NRF1, NRF2, Tfam, hepatic mRNA level of PGC1-alpha, and plasmatic level of glucose-6-phosphate [20], we reasoned that IF associated with HIIE could have an additive or a synergistic effect in energy metabolism involving mitochondrial reprogramming and hexokinase (HK) modulation. The activity of HK is closely connected with mitochondrial activity since the muscular isoform of this enzyme can bind to the mitochondrial membrane through VDAC [21]. This binding is described to positively modulate the activities of both HK and VDAC [22]. Additionally, we centered our assessment on HK, as this enzyme is known to be essential for overcoming the rate-limiting steps of glucose metabolism [23] and oxidative phosphorylation [24].

## Material and methods

### Animals and intermittent fasting protocol

All animal procedures performed received prior approval from the Animal Use Ethical Committee in the Health Science Center of the Federal University of Rio de Janeiro (Rio de Janeiro, RJ, Brazil; Protocol CEUA/EEFD06). At the beginning of the adaptation phase, twenty-four (6 animals/group) 60-day-old male Wistar rats weighing 262 ± 22 g were obtained from Biotério Central (Health Science Center of the Federal University of Rio de Janeiro) and housed in a climate-controlled environment (22.8 ± 2.0 °C, 45–50% humidity) with a 12/12–light/dark cycle with access to food and water *ad libitum*. Three weeks before the beginning of the study, animals from IF, HIIE and IF/HIIE groups were acclimated to the experimental protocols: Two weeks under the IF regimen (IF and IF/HIIE groups) followed by one week with the IF regimen plus HIIE (no overload, HIIE and IF/HIIE groups). The chow given to the animals was a standard laboratory chow Nuvilab CR-1 (Nuvital Nutrientes, Paraná, Brazil) with 22% protein, 8% fibers, and 4% fat. Animals in the control (C) and HIIE groups had access to food *ad libitum* during all the study while those in the IF and IF/HIIE groups were subjected to an every-other-day IF regimen. The IF and IF/HIIE groups were provided access to food *ad libitum* for 24 hours that was alternated with 24 hours without food. Animals were weighed weekly in the morning before the withdrawal or reintroduction of food. Food consumption was evaluated daily and the intermittent fasting resulted in a 15% reduction in total offered calories. Importantly, at the beginning of the study, the animals had already reached 90 days of age (young adults), thus avoiding influences of sexual maturation (30–40 days of age) [25] and musculoskeletal development [26] in our analyzes.

### High intensity intermittent exercise protocol and physical tests (PTs)

**Fig 1:**
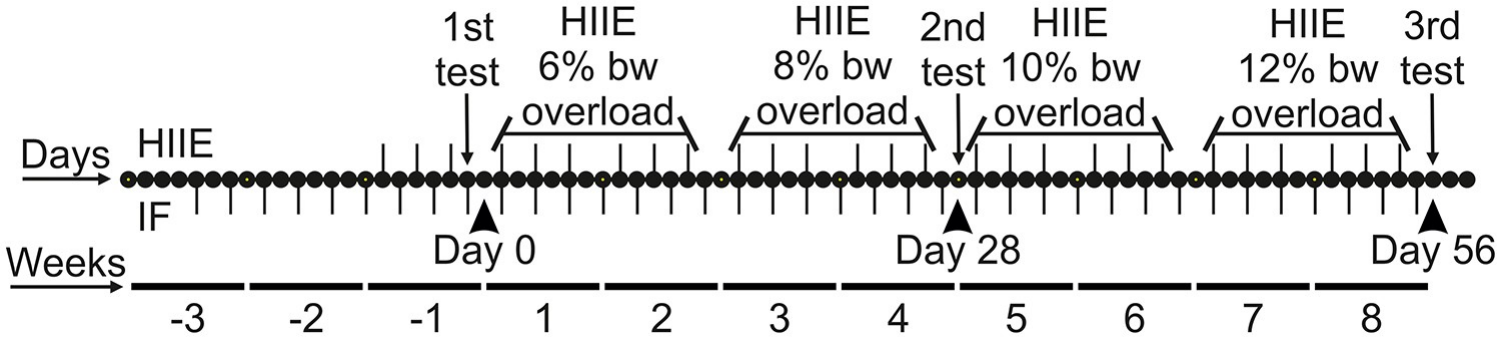
Graphical representation of the experimental design. Days (dots) and weeks (horizontal lines) of the study period (56 days in total) showing the exact days of fasting (lower dash) and HIIE (upper dash) interventions. The load utilized for each HIIE is described above the respective days. The 1st, 2nd, and 3rd PTs for endurance are indicated. The adaptation phase is represented by weeks with negative numbers.

The groups HIIE and IF/HIIE performed 8 weeks of an interval swimming exercise consisting of 14 repeated 20-second swimming bouts with weight (equivalent to percent body weight (bw)) attached with 10 seconds rest between the repeats as described previously [27]. Before the beginning of the study, animals were adapted one week to the aquatic conditions by performing the exercise without an overload. An initial overload of 6% of the bw was attached to the animal during the swimming period. The load was increased by 2% bw every two weeks. The HIIE protocol was performed exclusively on Mondays, Wednesdays and Fridays during the protocol adaptation and continued during the 8 weeks of the study. This routine was employed to avoid overtraining the animals. PTs were used to determine changes in cardiorespiratory endurance in the animals. Each test consists of the measurement of swimming until fatigue time under a 12% bw overload, that was applied on three separate occasions: (1st) one day before day 0, (2nd) at day 28, and (3rd) at day 56 (Fig 1). Fatigue was characterized when the animal failed to rise to the water surface to breathe within a 12 second period (counted with a chronometer when the animal was 10 cm below the surface).

### Intraperitoneal glucose tolerance tests (IGTT)

The animals were fasted for 12 hrs prior to the administration of an intraperitoneal injection of glucose (2 g/kg bw). Blood samples were drawn from the tail vein immediately before the glucose challenge, as well as 15, 30, 60, 90, and 120 min thereafter. Blood glucose levels were determined using an Accu-Chek glucose analyzer (F. Hoffmann-La Roche Ltd, Basel, Switzerland). The area under the curve (AUC) was calculated from glycemic curves over time using Prism 7.0 (Graph Software Inc., La Jolla, CA, USA).

### Fasting plasma insulin evaluation

Blood samples were collected in 8h fasted animals with heparinized tubes and the plasma was separated by centrifugation and kept in -80 °C. The total insulin level of the frozen plasma samples was measured using an Elisa-based method by VetLab Veterinary Clinical Pathology Laboratory (Petropolis, Rio de Janeiro, Brazil).

### Tissue collection and preparation

**Fig 2:**
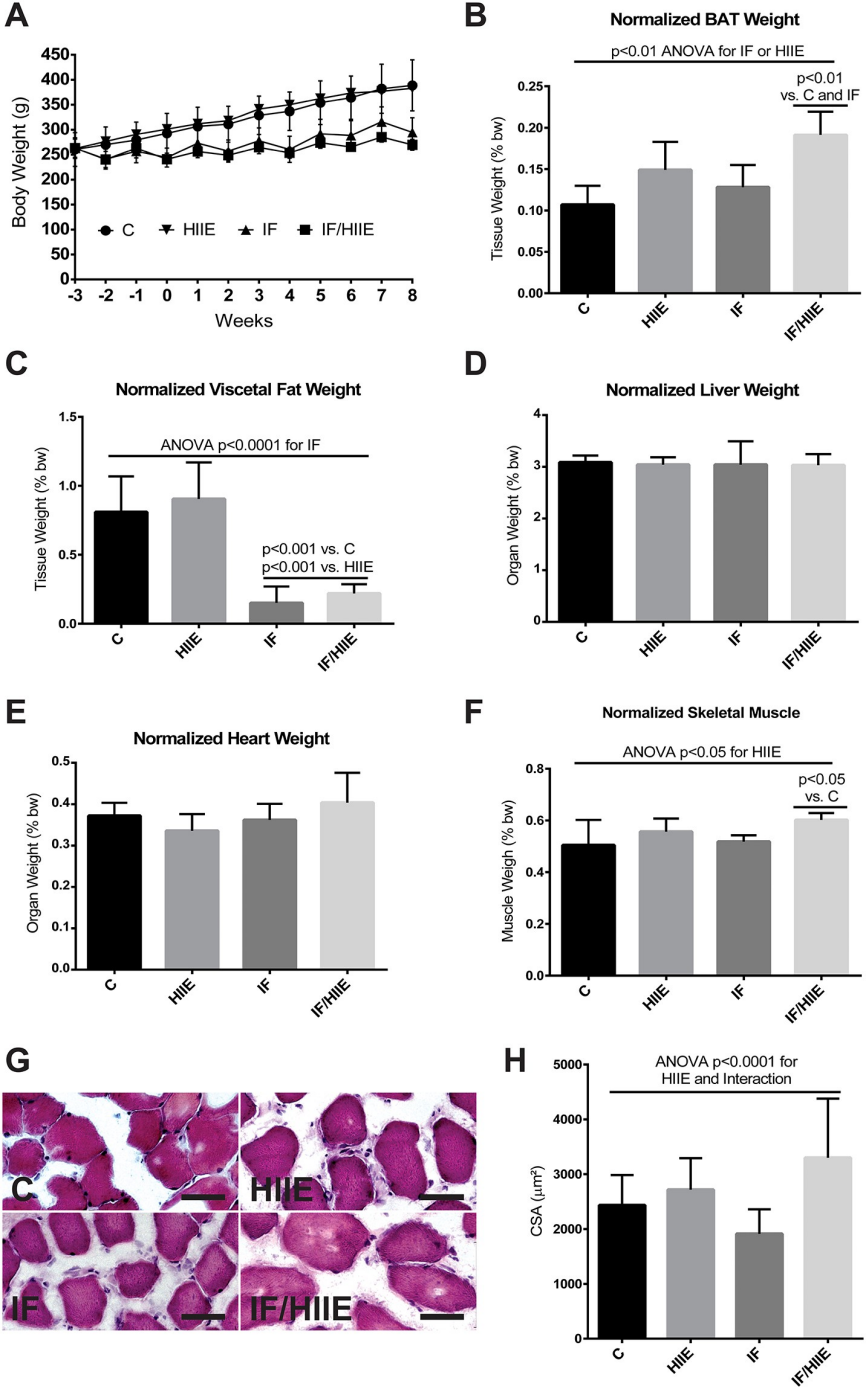
IF/HIIE prevents weight gain and adiposity and increases skeletal muscle cross-sectional area. (A) The bw curve from the weight weekly obtained in the morning before the withdrawal or reintroduction of food. Tissues and organs were collected from animals 2 days after day 56, the end of the study period, and immediately weighed. (B) normalized brown adipose tissue weight; (C) normalized visceral fat weight; (D) normalized liver weight (intact organ); (E) normalized heart weight (intact organ); (F) normalized skeletal muscle weight (gastrocnemius); (G) cross-sectional area histological profile (myofibers were stained with HE; 50 μm bar) and (H) quantification (columns with mean values and SD bars of 150 myofibers per animal (n = 3) per group). All tissue weights are normalized by the last bw measured before euthanasia. Each bar represents the mean ± SD; A-F) n = 6; H) n = 3. Individual effects (IF and HIIE) and the effect of their interaction were verified by two-way ANOVA and multiple comparisons; p values were annotated.

Heart, liver, gastrocnemius muscle, brown adipose tissue and visceral fat were rapidly removed from animals euthanized by decapitation 2 days after the last day of the experimental period (56 days) and weighed. All organs and tissues weights were normalized by the total bw (last bw measured before euthanasia) of the animals of origin (Fig 2). For enzymatic analysis and NADH measurements, tissues were homogenized with a Potter-Elvehjem in homogenization buffer (30 mM KCl, 4 mM EDTA, 250 mM sucrose, and 100 mM Tris-HCl (pH 7.5) with the protease inhibitors aprotinin and PMSF). Homogenates were centrifuged (5000 x g for 10 min at 4 °C) to obtain the supernatants that were maintained at 4°C. For the respiration analyses, the gastrocnemius muscle was minced and transferred to ice-cold BIOPS buffer (10 mM Ca^2+^/EGTA, 0.1 mM free Ca^2+^, 20 mM imidazole (pH 7.1), 50 mM K^+^-MES, 0.5 mM DTT, 6.56 mM MgCl_2_, 5.77 mM ATP, 15 mM phosphocreatine). Next, saponin (50 μg/ml) was added and incubated for 30 min at 4 °C, followed by a buffer exchange into ice-cold BIOPS without saponin. Samples were further incubated for 2h at 4 °C prior to high-resolution respirometry experiments.

### Determination of fiber cross-sectional area

Frozen gastrocnemius histological sections (5μm) were obtained in Leica CM 1850 Cryostat (Leica Biosystems, Nussloch, Germany), fixed with 4% formal calcium and stained with the hematoxylin and eosin (HE) method. We captured images of the stained sections with a Leica DM 2500 optical microscope (20 x lens) (Leica Biosystems, Nussloch, Germany). One hundred and fifty myofibers from 3 fields (50 fibers per field) per animal (n = 3) per group were selected and the fiber cross-sectional area was measured using Image J 1.51n software (NIH, Bethesda, MD, USA). A total of 150 myofibers were plotted for each animal to provide a reasonably reliable estimate of the total fiber number [28].

### Hexokinase activity

The method to measure enzymatic activity was adapted from one described previously [29]. The enzymatic activity was performed at 37 °C in a buffer containing 4 mM MgCl_2_, 50 mM Tris-HCl (pH 7.5), 20 mM glucose (0.1 mM glucose for liver), 4 mM ATP, 1 U/ml G-6PDH, 0.5 mM β-NADP^+^, and 0.1% Triton X-100 with a protein concentration of 0.05 mg/ml. The absorbance at 340 nm was acquired every 30 seconds for 30 minutes and the enzymatic activity was calculated using a molar extinction coefficient of 0.00622 uM^-1^ cm^-1^ for NADPH.

### Oxidative profile measurements

The oxidative profile of skeletal muscle was determined through the measurement of its mitochondrial NADH content by its fluorescence (reviewed in Mayevsky and Barbiro-Michaely [30]). Briefly, 140 μg of protein from a muscle sample homogenate derived from each animal was placed into a 96 well plate and excited at 340 nm. The emission at 450 nm was measured in a Spectramax Paradigm (Molecular Device, Sunnyvale, CA, USA). The assay was repeated three times and the fluorescence values were plotted as arbitrary numbers.

### High resolution respirometry

Respiration measurements were performed on fiber bundles in 2 ml of mitochondrial respiration medium 05 (110 mM sucrose, 60 mM potassium lactobionate, 0.5 mM EGTA, 3 mM MgCl_2_, 20 mM taurine, 10 mM KH_2_PO_4_, 20 mM HEPES (pH 7.1), 2 mg/ml BSA). O_2_ consumption was measured using the high-resolution Oxygraph-2k system (Oroboros Instruments GmbH, Innsbruck, Austria). The results were normalized to the wet weight of the permeabilized fiber bundles. All the experiments were performed at 37 °C in a 2 ml chamber. Mitochondria membrane permeability was tested by the addition of 10 μM cytochrome c. No greater than a 10% increase in oxygen consumption was observed. Multi-substrate titrations, respiratory states and respiratory control ratio calculations were performed. State 3 was measured after addition of complex I substrate, complex II substrate, and ADP. State 4o was measured subsequently to State 3 after addition of oligomycin to mimic State 4. State uncoupled was measured in the sequence through addition of FCCP. RCR was calculated by State 3 divided by State 4o [31].

### FoF1 ATP synthase activity assay in skeletal muscle

The ATP synthase activity was extrapolated from ATP hydrolysis (ATPase) activity described previously [32]. Briefly, 50 μg of protein from gastrocnemius homogenate from each animal was used to measure ATPase activity in 1 mM ATP, 5 mM MgCl_2_, and 50 mM Tris (pH 8,5) buffer in the presence or absence of 5 mM sodium azide at 37 °C. After TCA precipitation (20% w/v) and centrifugation (3000 x g for 15 minutes at 4 °C) the resultant supernatant was collected and combined with ammonium molybdate and Fiske-Subbarow reducer. The absorbance was measured at 660 nm.

### Protein oxidation

Protein oxidation levels were measured in plasma samples using the protein carbonyl content method, as previously described [33]. Briefly, the blank sample was mixed with 2.5 N HCl and the other with 2,4-dinitrophenylhydrazine (freshly prepared in 2.5 N HCl) and the resulting solutions were incubated in the dark for 1 h at RT with intermittent vortexing (every 15 min), with subsequent addition of 10% TCA (w/v). After centrifugation, the pellet was washed once with 10% TCA and three times with ethanol: ethyl acetate (1:1 v/v). The resulting pellets were suspended in 5 M urea (pH 2.3), incubated at 37 °C for 15 minutes and centrifuged at 15000 x g for 5 minutes. The resulting supernatant absorbance was determined at 370 nm, and results were expressed as nmol carbonyl / mg protein.

### Lipid peroxidation

Lipid peroxidation levels were measured using the thiobarbituric acid method (TBARS), with minor modifications of the technique previously described [34]. Plasma samples were diluted in 100 mM sodium phosphate buffer (pH 7.4), 1:3 (v/v), with subsequent addition of cold 10% TCA and kept on ice for 15 minutes. Afterwards, samples were centrifuged at 2200 x g for 15 min (4 °C) and to the resultant supernatants were added equal volumes of 0.67% thiobarbituric acid (w/v) followed by a water bath (95 °C) incubation for 2 h. After cooling, the absorbances were read at 532 nm in a 96-well plate reader, Spectra Max Paradigm (Molecular Devices, California, United States). Results were expressed in μM malondialdehyde (MDA).

### Statistical analysis

Comparisons were performed using two-way ANOVA with Tukey’s multiple comparison test. Data are presented as mean ± standard deviation (SD) and p values < 0.05 were considered significant. All statistical analyses were performed using Prism 7.0 (Graph Software Inc., La Jolla, CA, USA).

## Results

### The effect of IF and HIIE on body composition

To determine the adaptive changes on energetic metabolism and physical performance induced by IF, HIIE, and their combination (IF/HIIE), the three regimens were imposed on age-matched young adult Wister rats over 8 weeks (Fig 1). Over the course of the experimental conditions, the weight of the animals was tracked weekly and plotted in a curve (Fig 2A). The cumulative increase in the weight of animals on an *ad libitum* diet (C and HIIE groups) is very apparent and the prevention of excessive weight gain in animals under IF protocol (IF and IF/HIIE groups) is evident; other groups observed a similar effect [12, 18]. To explore the possible origin of the observed differences in the weight of the groups, organs and tissues were collected and weighed at the end of the study. The organs and tissue weight values were normalized by total body mass of the animal of origin. Initially, we weighed the brown adipose tissue (BAT, Fig 2B) and the visceral fat (Fig 2C) of the animals. Our results demonstrate that IF combined with HIIE can induce an increase in BAT mass; the statistical evaluation pointed to a contribution of both IF and HIIE in the result. Differently, IF alone was responsible for the lower visceral fat weight. The latter was also observed by Li et al. [12]. No modifications were observed in the liver (Fig 2D) and heart (Fig 2E) normalized weights. An increase in skeletal muscle mass (gastrocnemius) (Fig 2F) was observed in IF/HIIE. In this case, only the HIIE contribution was observed. Interestingly, the evaluation of cross-sectional area (CSA) confirmed the hypertrophic effect of HIIE (Fig 2F and 2G), but also demonstrate a clear synergistic effect of IF/HIIE (ANOVA p < 0.05 for interaction), while the measurement of CSA from IF demonstrates an atrophic tendency (p = 0.08).

### IF/HIIE impact on glucose metabolism

**Fig 3:**
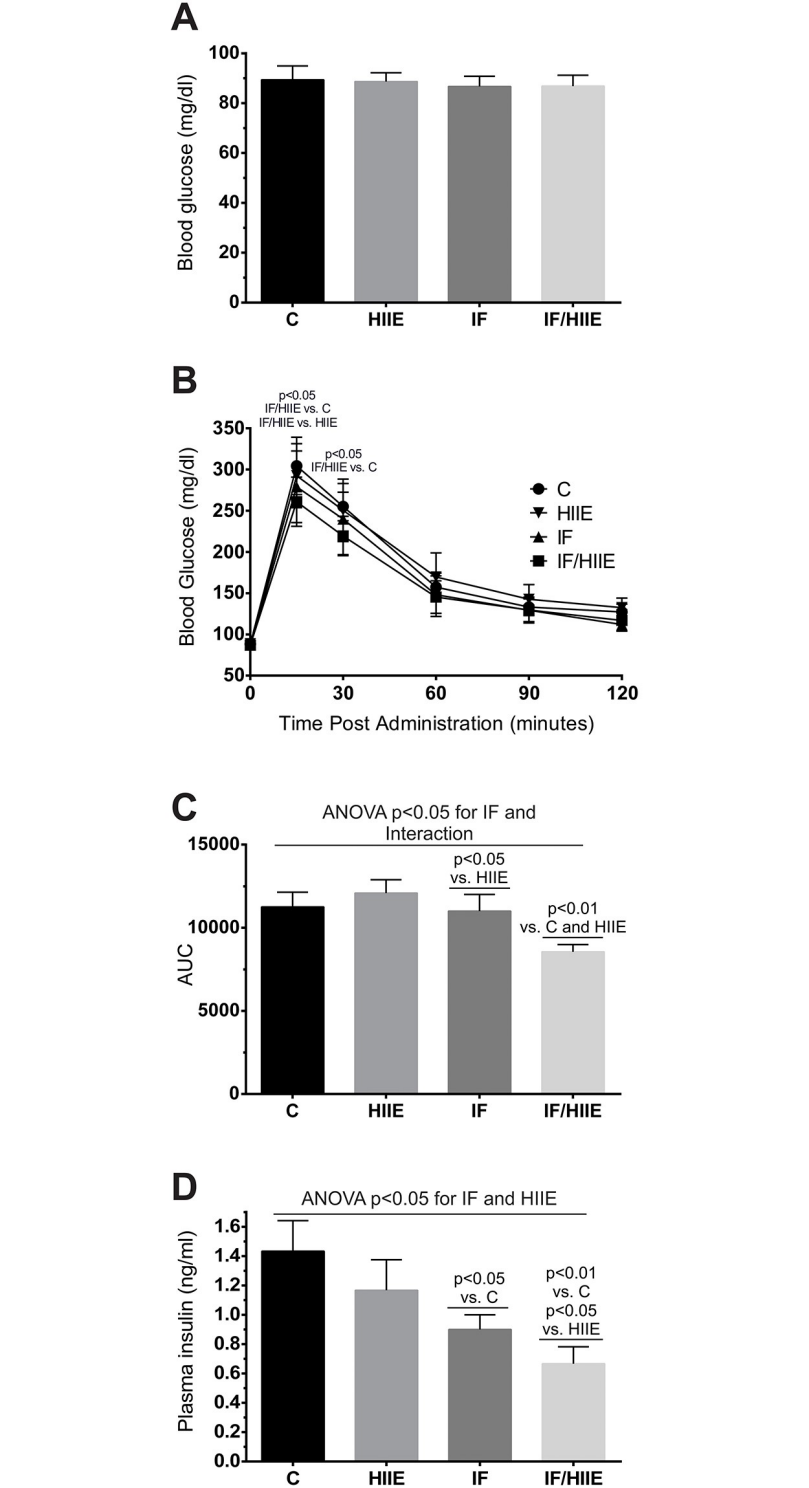
IF/HIIE present enhanced glucose tolerance and lower levels of fasting plasma insulin. (A) Plasma blood glucose levels. (B) IGTT. Animals were fasted for 12 h prior to the administration of an intraperitoneal injection of glucose (2 g/kg bw). (C) AUC calculated from IGTT curve (B). (D) Insulin plasma levels of fasted animals were measure using an Elisa-based method. Each bar represents the mean ± SD; A-D) n = 6. Individual effects (IF and HIIE) and the effect of their interaction were verified by two-way ANOVA and multiple comparisons; p values were annotated.

The effect observed above in the body composition could be explained by an intensive reprograming of energetic metabolism. To investigate the effects of IF, HIIE and the combination of both on energetic metabolism, we initiated the measurement of general indicators of glucose metabolism and predictive factors for insulin sensitivity [35, 36]. We measured the fasting blood glucose levels, glucose tolerance (IGTT) and fasting plasma insulin levels (Fig 3). All groups presented similar values for fasting blood glucose (Fig 3A) and the glucose tolerance test showed some dissimilarities between groups, most notably before 60 min (Fig 3B). To better scrutinize the glucose tolerance curves, we measured the AUC, revealing that IF/HIIE poses an enhancement of glucose tolerance (Fig 3C). The statistical evaluation revealed a contribution of IF along with the contribution of an interaction between IF and HIIE, evidencing a synergistic effect. In contrast, the fasting insulin level (Fig 3D) experienced an influence mainly by IF with an additive effect of HIIE. The additive effect observed in IF/HIIE was reflected in the fasting insulin numbers: 0.66 ± 0.08 ng/ml for IF/HIIE versus 1.43 ± 0.15 ng/ml for C.

**Fig 4:**
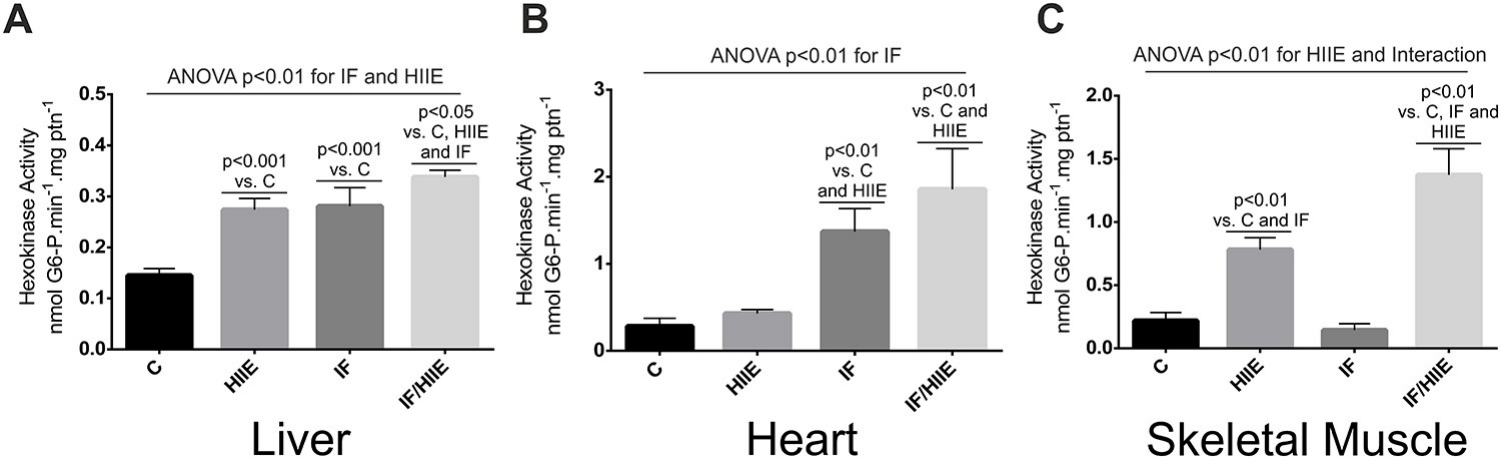
Synergistic effect of IF/HIIE on low Km HK activity. The enzymatic activity was calculated from the NADPH production. (A) HK activity in the liver. (B) HK activity in the heart. (C) HK activity in the skeletal muscle (gastrocnemius). Each bar represents the mean ± SD; A-C) n = 6. Individual effects (IF and HIIE) and the effect of their interaction were verified by two-way ANOVA and multiple comparisons; p values were annotated.

By treating rat thymocytes with 2-deoxy-D-glucose, phorbol and mannoheptulose, Naftalin and Rist demonstrated that HK activity is integrated with the sugar transporter [37]. Based on this mechanism, we hypothesized that the enhanced glucose tolerance combined with low insulin levels observed above could be, in part, explained by an augmented HK activity in multiple organs (Fig 4). To test this we aimed to evaluate the activity of low Km isoforms of HK (I and II) present in the liver, heart and gastrocnemius [38, 39]. In the liver, both IF and HIIE individually promoted activation of the catalytic HK activity (Fig 4A), the combination of IF and HIIE produced a slightly enhancement observed in the IF/HIIE group. In the heart, the main effect in HK catalytic activation was produced by IF alone (Fig 4B). Strikingly, in the skeletal muscle (Fig 4C), HIIE promoted a strong contribution for HK activity (3-fold increase in HIIE), but the interaction maximized the catalytic activation (6-fold increase in IF/HIIE). The latter reaffirmed the synergic effect from the association between IF and HIIE. Aiming to test the above proposed correlation between HK activity with glucose tolerance values (IGTT—AUC), we plotted the individualized values for HK for each animal separately by organ (and tissue) against the AUC value from the same animal and we found an indication of inverse correlation in all the organ/tissue evaluated (S1 Fig).

### IF/HIIE synergistic effect in physical activity and energy production

**Fig 5:**
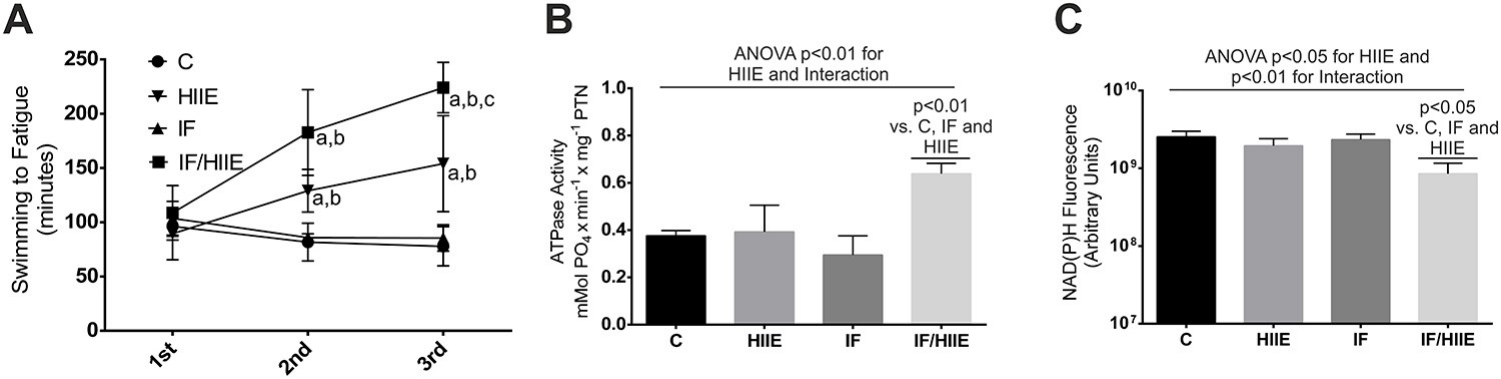
PT and energy production. (A) PT. The physical endurance was measured using swim until fatigue test (under a 12% bw overload). The tests were applied on three separate occasions: (1st) one day before day 0, (2nd) at day 28, and (3rd) at day 56. p < 0.05; a vs. control, b vs. HIIE, and c vs. IF. (B) FoF1 ATP synthase activity was estimated through the ATPase activity of the enzyme. (C) NAD(P)H autofluorescence was measured directly in fresh muscle homogenates (gastrocnemius). The excitation and emission wavelengths were 340 nm and 450 nm, respectively. Each bar represents the mean ± SD; A-C) n = 6. Individual effects (IF and HIIE) and the effect of their interaction were verified by two-way ANOVA and multiple comparisons; p values were annotated.

The effect of HIIE on promoting physiological adaptation in skeletal muscle is well described [14, 40]. However, the effect of IF combined with HIIE in physical performance is not, although some reports that employed endurance training suggest a possible additive effect: I) Rodriguez-Bies et al. combined IF protocol with endurance exercises and observed a consistent increase in beta-oxidation, lactate production and mitochondria content in the gastrocnemius with a modest effect on physical performance in animals submitted to both protocols compared to the control group [41]. II) Moraes et al. showed a preserved muscle mass in animals submitted to an IF protocol coupled to endurance exercises [42]. However, the physical capacity of these animals was not evaluated in the latter. To investigate a possible improvement of physical endurance promoted by IF and/or HIIE along the experimental procedures, we submitted all groups to a PT on three days (Fig 5A): 1st) one day before day 0; 2nd) at day 28; 3rd) at day 56 (end of the IF and HIIE protocols). The 3rd test revealed a higher endurance of animals in the HIIE and IF/HIIE groups that was approximately 90% and 180% in comparison to the control group, respectively. The possibility that the weights of the animals from the IF and IF/HIIE groups were contributing to the outcome of the PT was eliminated since no correlation was observed between swimming time and the weights of the animals in any of the PTs (S2 Fig).

Furthermore, to explore any possible adaptation that granted the IF/HIIE group the best results in the PT, we measured the activity of the FoF1 ATP synthase in the skeletal muscle of these animals (Fig 5B). We observed approximately 50% increase in FoF1 activity in the IF/HIIE group compared to the other groups; contribution from HIIE and from interaction in the result were observed. According to the literature, both IF [41] and HIIE [43, 44] alone could promote adaptations in mitochondria. To have a general overview of mitochondria electron transport chain in skeletal muscle, we employed a comparative method that uses tissue NAD(P)H autofluorescence [30] to indirectly evaluate oxidation rate profile of the groups (Fig 5C). The bulk of the measurement was assumed to be from the mitochondria since cytosolic NADH and NAD(P)H contribute in general less than 20% of the signal under these conditions [45]. Only the IF/HIIE group presented statistically significant lower levels of NAD(P)H with a contribution from HIIE and from interaction in the result, like those observed in FoF1 activity (Fig 5B). The lower level of NAD(P)H measured in the IF/HIIE group suggests a higher mitochondrial oxidation rate in comparison with other groups. The effect of IF/HIIE interaction observed in FoF1 activity and mitochondrial oxidation rate assays corroborate the proposed synergistic effect of IF/HIIE in mitochondria energy production.

### Synergistic effect of IF/HIIE in muscle fiber mitochondria respiratory states

**Fig 6:**
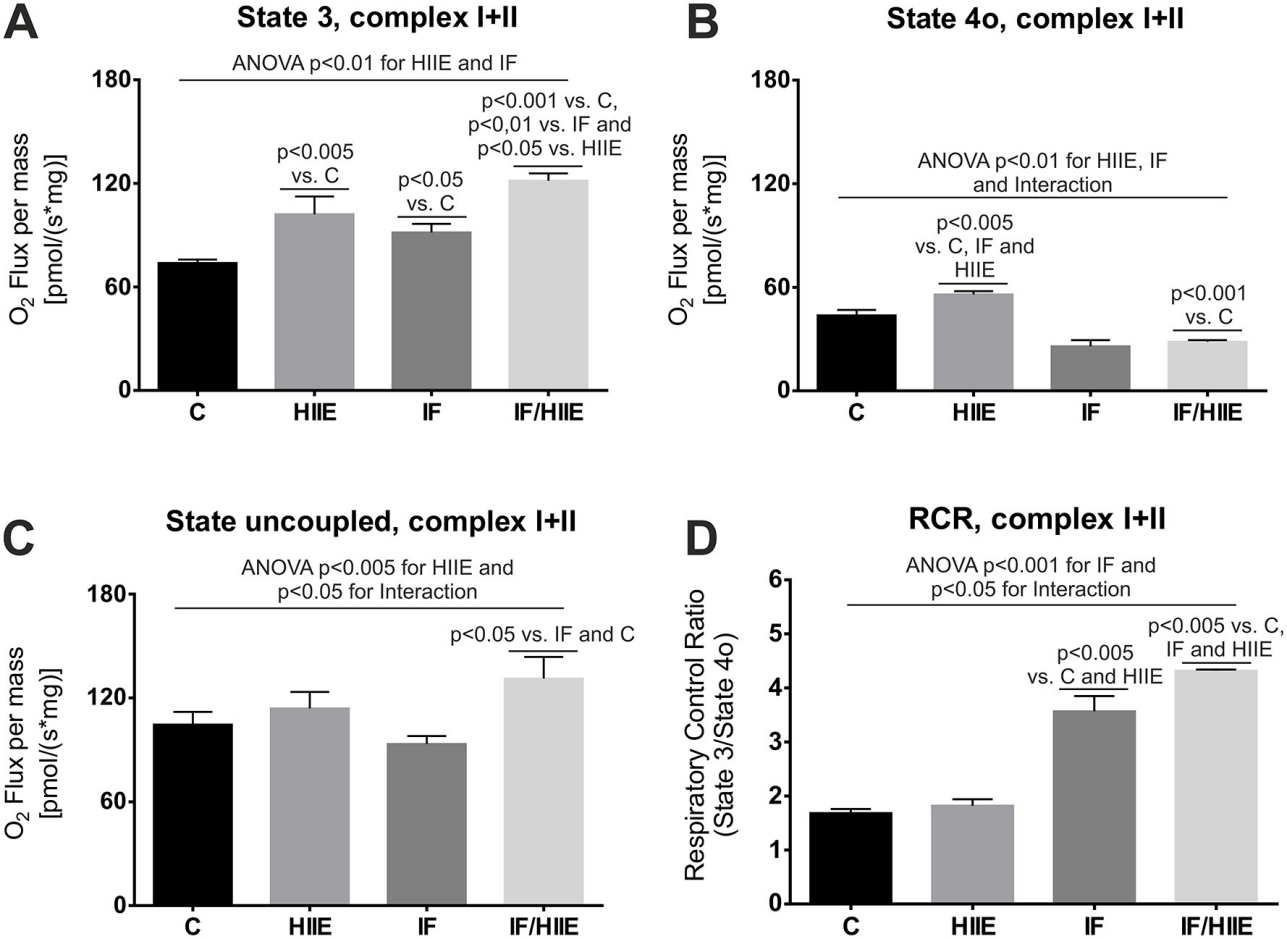
IF/HIIE promotes mitochondria activation in permeabilized skeletal muscle fiber bundles. Respiration measurements were performed on gastrocnemius fiber bundles using a high-resolution respirometry. The results were normalized to the wet weight of the permeabilized fiber bundles. (A) State 3 O_2_ flux. State 3 was measured after addition of complex I and II substrates and ADP. (B) State 4o O_2_ flux. State 4o was measured subsequently to the State 3 after addition of oligomycin to mimic real State 4 (depletion of ADP). (C) State uncoupled O_2_ flux. State uncoupled was measured in the sequence of state 3 and state 4o through addition of FCCP. (D) RCR calculation. RCR was calculated by State 3 divided by State 4o. Each bar represents the mean ± SD; A-D) n = 3. Individual effects (IF and HIIE) and the effect of their interaction were verified by two-way ANOVA and multiple comparisons; p values were annotated.

To investigate how the skeletal muscle mitochondrial respiratory complexes could be affected by the IF and/or HIIE protocols and to help understand the above results, we measured the different respiratory states and the mitochondria respiratory control rate (RCR) (Fig 6). RCR is an reliable indicator of mitochondrial function, as high RCR usually indicates healthy mitochondria and low RCR usually indicates mitochondrial dysfunction [46]. Initially, we analyzed muscle fiber mitochondria oxygen consumption in the presence of complex I substrate, complex II substrate, and ADP (State 3). We observed a higher O_2_ flux rate related to ATP production in the IF/HIIE group, followed by both the HIIE and IF groups individually, in comparison to the C group (Fig 6A). The individual contribution of IF and HIIE to the observed effect as well the additive effect of IF plus HIIE are evident. Subsequently, we added an ATP synthase inhibitor, oligomycin (State 4o). The HIIE group presented a higher O_2_ flux (Fig 6B), indicative of increased proton leakage and/or extra-mitochondrial O_2_ consumption. In contrast, the IF and IF/HIIE groups presented a lower O_2_ flux in comparison to the C group (Fig 6B). Next, FCCP was added to uncouple the O_2_ flux from ATP production (State uncoupled). The IF/HIIE group presented higher values in comparison with the other groups (Fig 6C), indicative of an augmented number of respiratory complexes and/or mitochondrial mass. The RCR calculated values were greatest in the IF/HIIE group followed by the IF group, both of which were significantly greater than the values of the C and HIIE groups that were nearly equal (Fig 6D).

The results above are indicative of an improved O_2_ flux and ATP production coupling in the IF/HIIE and IF groups. The respiratory profile of the IF/HIIE group resembles data from a different group that showed an augmented mass of mitochondrial in skeletal muscle using a dissimilar exercise protocol [41]. Finally, the greater RCR value agrees with a more oxidative profile and active FoF1 synthase that was observed exclusively in the IF/HIIE group. Additionally, the values observed in State 4o, State uncoupled and RCR for the IF/HIIE group mitochondria clearly demonstrate an IF/HIIE interaction effect on these results that corroborate the proposed synergism of IF and HIIE.

### Overall oxidative stress markers are reduced in the IF/HIIE group

High intensity exercise was clearly demonstrated to negatively modulate the muscular redox state with a resultant increase in lipid peroxidation [47]. However, the adoption of HIIE models was also shown to induce positive effects in muscular physiology (reviewed in MacInnis and Gibala [48]). We hypothesized that in addition to local effects promoted by HIIE in skeletal muscle, this regimen could also affect the overall redox state with a modulation in oxidative stress markers and, in combination with IF, could possibly generate an additive or a synergistic effect.

**Fig 7:**
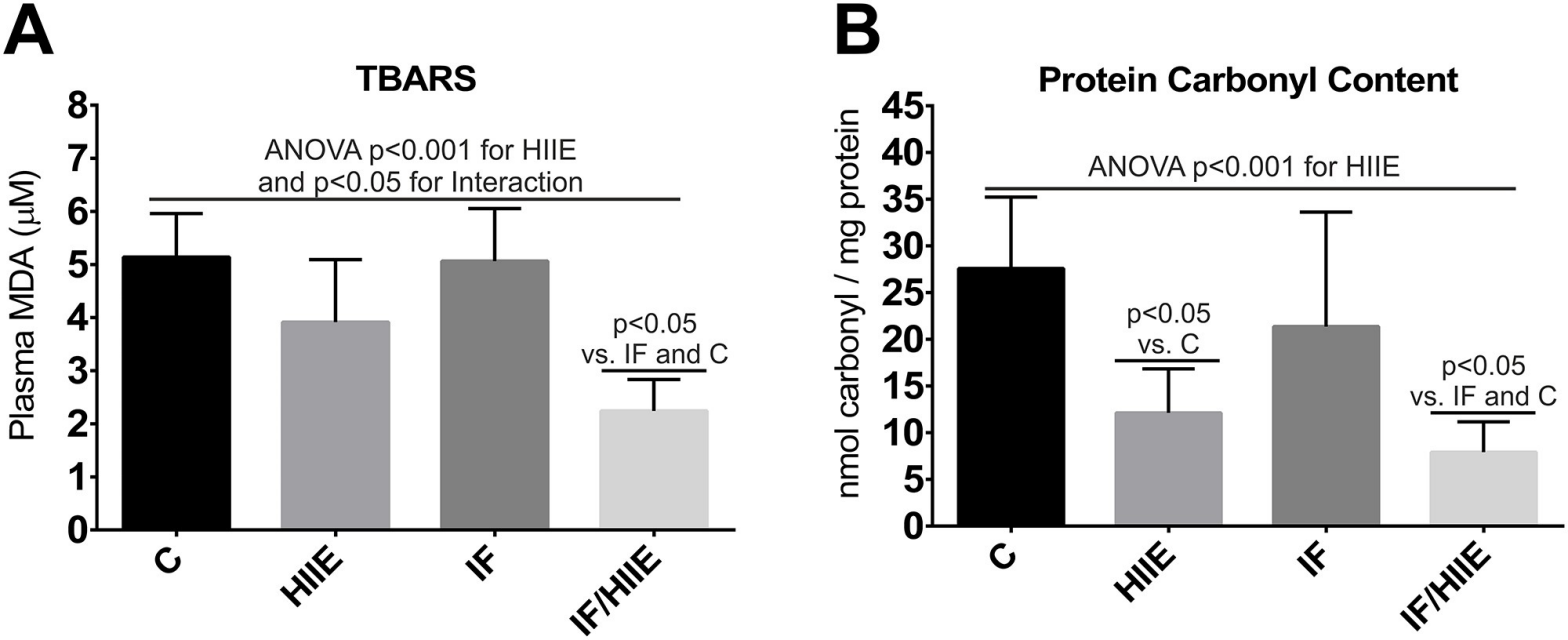
The IF/HIIE group presented lower levels of plasma lipid peroxidation and protein oxidation. Oxidative damage markers were measured in the plasma through quantification of (A) lipid peroxidation (MDA) and (B) protein oxidation (protein carbonyl content). Each bar represents the mean ± SD; A-C) n = 6. Individual effects (IF and HIIE) and the effect of their interaction were verified by two-way ANOVA and multiple comparisons; p values were annotated.

To investigate the effect of IF and HIIE in the overall redox state, we measured plasma oxidative stress damage markers level: lipid peroxidation and protein oxidation through malondialdehyde (MDA) quantification and protein carbonyl content, respectively. We observed a strong reduction in MDA levels in the IF/HIIE group (Fig 7A); contribution of HIIE and IF/HIIE interaction was detected in the result. The latter suggests a synergism between IF and HIIE in lipid peroxidation prevention. Furthermore, the IF/HIIE group presented lower levels of plasma protein oxidation followed by the HIIE group (Fig 7B). The statistical analysis demonstrates a strong contribution of HIIE with a small additive effect of IF in the result. The global observation of oxidative stress markers reveals that IF/HIIE could promote synergistic (Fig 7A) and additive (Fig 7B) effects.

## Discussion

Over the last years, IF [10, 49] or HIIE [50, 51] protocols were individually evaluated for their potential to promote energy metabolism and physiologic adaptations. Different combinations of fasting regimens with a variety of exercise protocols were also employed [52], however, none of these studies has examined the interaction between IF and HIIE accurately. Recently, researchers have begun to dissect the mechanisms underlying IF and HIIE adaptations: For IF, some of the key changes are high circulating levels of acetate (serum) combined with genetic reprograming of white adipose tissue (*UCP1*, *MCT1* and *Glut4*) [12], whereas for HIIE a proteomic change has been proposed for skeletal muscle cells that are especially noticeable in mitochondria and ribosome protein profiles [53]. These discoveries shed new light on IF and HIIE, thus motivating us to investigate a potential additive effect from the combination of these strategies.

Aiming to generate as little impact in the animals’ stress level as possible, and to avoid an initial overeating (IF group—non-fasting day) impact on exercise execution, we initially chose to include a long adaptation time for the distinct protocols, thus avoiding any misleading precipitate result. Afterwards, the protocols were maintained for 2 months, giving us the opportunity to observe some important effects that were not observed in a concurrent work published recently [20]. We also evaluated the relative contribution of each protocol (IF and HIIE) in our results and surprisingly identified synergism between the protocols in addition to the additive effects published elsewhere.

We identified different contributions of IF or/and HIIE in the body composition. IF was mainly responsible for lowering visceral fat. On the other hand, HIIE was mainly responsible for increasing skeletal muscle mass. These IF and HIIE individual effects are well established, however, the combination of both promoted a synergistic effect: BAT and skeletal muscle hypertrophy (CSA). The synergistic effect of IF/HIIE in improving skeletal muscle hypertrophy (mass and CSA) was completely unexpected, in contrast to the effect on BAT that was already observed in animals under IF in combination with other exercise protocol [42].

Interestingly, BAT and skeletal muscle are very responsive to variations in plasma glucose and insulin levels [54, 55]. This means that understanding the IF/HIIE effects on glucose metabolism could give us a hint on the source of the observed adaptation in the body composition. However, the fasting blood glucose values from our animals remained largely unaltered (~88 mg/dl), in contrast to the data from the concurrent group using IF/HIIE [20]. Their groups presented blood glucose levels very different from our measurements: C and HIIE with ≥ 140 mg/dl, IF with ≤ 130 mg/dl, and IF/HIIE with ≤ 120 mg/dl. These divergent values complicate the comparison between our works. Next, we applied IGTT to evaluate peak plasma glucose elevation and recovery of glucose levels in the different groups. The IF/HIIE group had the better result in IGTT, emphasized by the AUC measurement that revealed a synergism between IF and HIIE. A similar IGTT result was obtained by Marosi et al. [20]. Additionally, we measured the fasting plasma insulin levels and observed a strong decrease of insulin levels in the IF/HIIE group (53% reduction), resulting from an additive effect between IF and HIIE.

To investigate the consequences of the observed modulation in glucose metabolism combined with low levels of circulating insulin, we measured the activity of the low Km HKs, whose expression are not controlled by feed/starve state, like HK IV [38]. We observed an additive effect of IF and HIIE in liver HK and in heart HK activity enhancement. In contrast, skeletal muscle HK activity was modulated by HIIE (3.5 fold) and interaction of IF/HIIE (6.2 fold), the latter corroborating the synergistic effect seen above. In our assay proposal and data interpretation, we assumed that the glucose transport was not impaired by any of our protocols, especially the Glut4 signaling that is described to be upregulated in IF and HIIE and in a series of variations of the latter protocols [56, 57].

The HK activity is closely related to mitochondrial activity, since the muscular isoform of this enzyme can bind to the mitochondrial membrane through VDAC [21], a binding that can positively modulate the activities of both [22]. In our work we measured the HK in a mitochondria-rich fraction, and we observed an increased HK activity combined with an increased FoF1 activity in skeletal muscle (gastrocnemius), culminating with the best result for IF/HIIE in the physical endurance test. Additionally, we interpreted the oxidative rate profiles comparing NAD(P)H autofluorescence in muscle homogenates from different groups and observed a higher oxidation rate in the IF/HIIE group. The latter could be explained by an increased activity of mitochondrial complex I, as partially demonstrated by Marosi et al. (by western blot) in their IF/HIIE group [20]. To measure the contribution of IF or/and HIIE on mitochondrial metabolism, we measured the oxygen consumption in the different groups in the following respiratory states: State 3, where statistical analysis revealed additive effect of IF and HIIE, and State 4o, State uncoupled and RCR, where statistical analysis revealed IF/HIIE interaction effect. This suggest that the IF/HIIE synergism can promote mitochondrial coupling and mitochondrial biogenesis in skeletal muscle. The indication of mitochondria coupling corroborates the observed FoF1 increased activity.

The correlation between mitochondria and free radicals is well established (review in Gutierrez et al. [58]). Additionally, we demonstrate that IF/HIIE synergism modulates mitochondria physiology, promoting more oxidative mitochondria. The latter effect could increase the generation of superoxide that can culminate in damage in lipids and proteins [59]. We observed similar patterns of result for the two oxidative damage markers (MDA and Carbonyl), the overall result reflects the cumulative adaptations promoted by HIIE that can be furthered by combination with IF promoting synergistic or additive effects.

To further discuss the adaptations promoted by IF/HIIE, as described above, that produced animals with low insulin levels, BAT and skeletal muscle hypertrophy as well as highly active skeletal muscle HK and indications of more coupled mitochondria, we should include in the equation the new role of myostatin observed in metabolism control. The tissue specific blockage of activin/myostatin axis in rats promoted the development of low insulin level in these animals accompanied with resistance to fat accumulation [60]. An unsuspected BAT-muscle crosstalk driven by myostatin was revealed recently in mice [61]. Additionally, treatment of cancer cells with myostatin promoted VDAC1 upregulation combined with downregulation and mitochondrial dissociation of HK [62]. The growing number of different lines of evidence has put the activin/myostatin axis onto our radar for investigation and we should address their role in IF/HIIE adaptation in the near future.

Finally, though the data presented here, the IF/HIIE protocol arises as a reasonable approach to be employed for translational investigation in humans, especially when considering the evolutionary-based proposal to combine IF and HIIE to mimic erratic food behavior and occasional high-intensity energy demand of the gathering/hunting activities of our ancestors to improve metabolism. Different factors influence exercise tolerance and fasting allowance in human population (especially with relation to age and health status), thus HIIE and IF protocols must be adapted accordingly.

## Supporting information

S1 Fig Analysis of the correlation between HK activity and IGTT—AUC. Individual values correlating HK activity and IGTT- AUC for each animal separately by organ (and tissue) were plotted and the correlations were analyzed and indicated in the figures together with the interpolation curve (mean and 95% confidence band).
(TIF)

S2 Fig Analysis of the correlation between animal weight and the swimming time to fatigue in each of the three PTs. Individual values correlating weight and swimming time to fatigue for every animal obtained in each PT day were plotted and the correlations were analyzed and indicated in the figures.
(TIF)
