# Supplementary figures and images for: The synergism of high-intensity intermittent exercise and every-other-day intermittent fasting regimen on energy metabolism adaptations includes hexokinase activity and mitochondrial efficiency
Source: PLoS One. 2018 Dec 21;13(12):e0202784. doi: 10.1371/journal.pone.0202784 (PMC6303071; doi:10.1371/journal.pone.0202784)

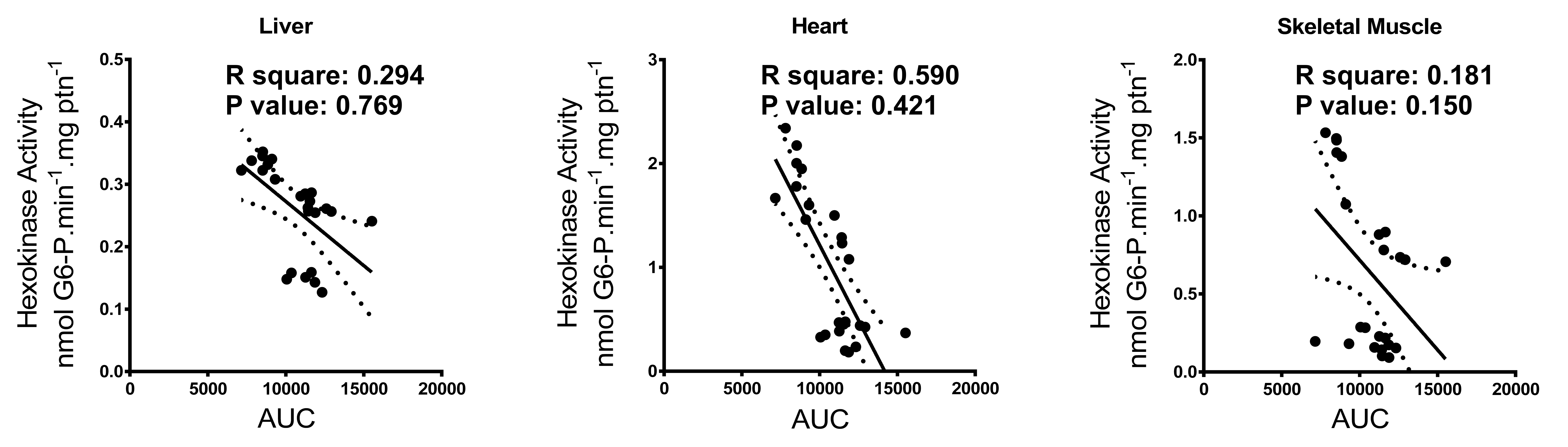

Supplement: S1 Fig — Individual values correlating HK activity and IGTT- AUC for each animal separately by organ (and tissue) were plotted and the correlations were analyzed and indicated in the figures together with the interpolation curve (mean and 95% confidence band). (TIF) [file pone.0202784.s001.tif]

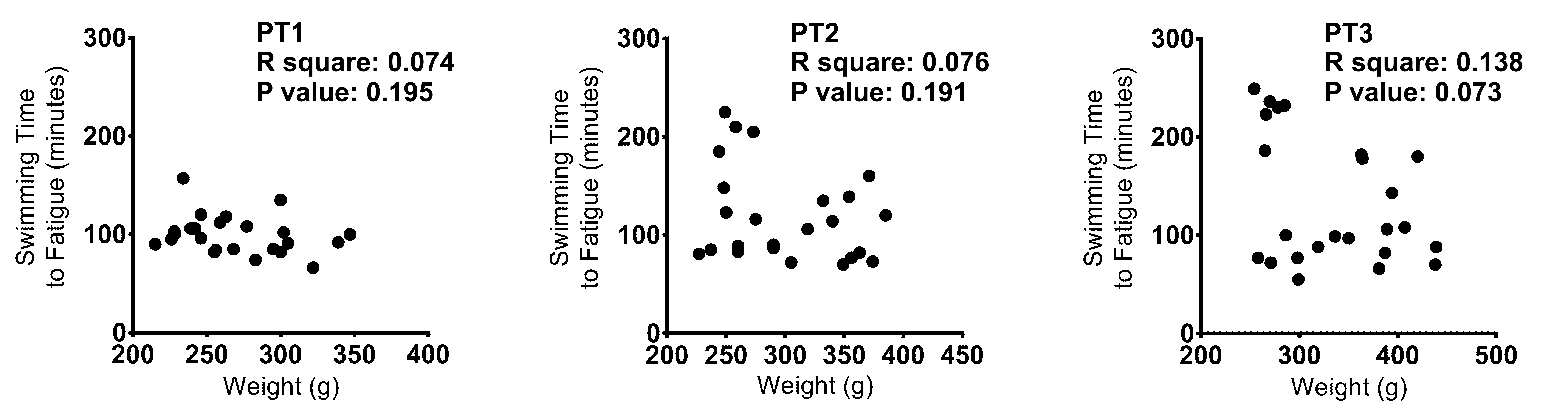

Supplement: S2 Fig — Individual values correlating weight and swimming time to fatigue for every animal obtained in each PT day were plotted and the correlations were analyzed and indicated in the figures. (TIF) [file pone.0202784.s002.tif]
